# Proposed Models for Prediction of Mortality in Stage-I and Stage-II Gastric Cancer and 5 Years after Radical Gastrectomy

**DOI:** 10.1155/2022/4510000

**Published:** 2022-03-08

**Authors:** Tianyi Fang, Xin Yin, Yufei Wang, Lei Zhang, Xinghai Zhang, Xudong Zhao, Yimin Wang, Yingwei Xue

**Affiliations:** ^1^Department of Gastroenterological Surgery, Harbin Medical University Cancer Hospital, Harbin Medical University, Harbin 150081, China; ^2^Department of Pathology, Harbin Medical University, Harbin 150081, China

## Abstract

The current American Joint Committee on Cancer (AJCC) staging system provides limited information for patients with early death from stage-I and stage-II gastric cancer (GC) and death at >5 years after radical gastrectomy. The aim of this study was to construct nomogram models to predict the mortality risk of these patients. In this study, clinical and pathological data on patients who underwent curative gastrectomy at Harbin Medical University Cancer Hospital between 2000 and 2014 were retrospectively collected. Receiver operating characteristic analysis was used to screen for sensitive serum immune biomarkers to predict the risk of mortality death >5 years after radical gastrectomy (Group A) and risk of early death in stage-I and stage-II GC (Group B). The prediction model was constructed by combining serum immune markers with clinicopathological features by R Studio. We found that serum fibrinogen (F), systemic immune inflammation (SII), and pTNM stage were independent risk factors for prognosis in Group A (*P* < 0.05). F, SII, age, Borrmann type, and scope of gastrectomy were independent risk factors for prognosis in Group B (*P* < 0.05). The area under the curve of the predictive model in Groups A and B was 0.726 and 0.848, respectively. In conclusion, the predictive models of F and SII combined with clinicopathological features can predict high mortality risk in patients with stage-I and stage-II GC and >5 years after radical gastrectomy, which will contribute to the supplement of the traditional AJCC system and to individual survival prediction.

## 1. Introduction 

With the enhanced awareness of public health protection in developing countries, the incidence rate of gastric cancer (GC) has been decreasing gradually, but it is still the sixth highest incidence and third highest mortality rate of malignant tumors worldwide [1]. TNM staging and histological subtype classification according to tumor infiltration, regional lymph nodes, and distant metastasis are the conventional criteria for evaluating prognosis and guiding treatment after surgery [2]. Radical surgery is the basic method of comprehensive treatment for GC [3]. In addition to the molecular targeted therapy, National Comprehensive Cancer Network (NCCN) guidelines also support the routine use of postoperative adjuvant chemotherapy for patients with stages II–IV GC [4]. However, it is well known that clinical outcome can significantly vary among patients receiving similar treatment regimens for the same histological tumor stage. This situation indicates that although the traditional classification tools provide effective prognostic information, they are still unable to satisfy individual evaluation.

At present, it is generally believed that there are two limitations in the eighth edition of TNM staging system for GC [5]. Firstly, it provides deficient information for predicting recurrence and metastasis at 5 years after radical gastrectomy. Secondly, it has some defects in the individualized risk assessment of early recurrence and death in patients with stage-I and stage-II GC. Patients with survival time >5 years are often considered to have clinically complete tumor regression. The recurrence rate of these patients is considered to be ∼10% [6, 7], and further postoperative review and follow-up are often omitted. For patients with stages IA, IB and II of GC based on the Japanese Gastric Cancer Association (JGCA) guidelines, the 5-year survival rate can reach 91.5%, 83.6%, and 70.6%, respectively [8]. As a result, clinicians pay less attention to these patients postoperatively than those with advanced-stage GC and ignore the possibility of early recurrence. In view of these two kinds of recurrence, it is necessary to supplement the traditional classification system for individualized risk prediction.

Clinicians have also made efforts for individualized tumor monitoring and construction of predictive models [9]. However, it is difficult for predictive models only based on tumor-related indicators to achieve satisfactory clinical application after further verification. Tumor immunity has an important role in controlling tumor progression, and more studies have shown that tumor progression or recurrence depends not only on tumor characteristics, but also on the immune response in the tumor microenvironment and peripheral blood. It has been proposed that adaptive immune response may play a critical role in preventing tumor recurrence [10]. In particular, memory T cells in peripheral blood can disseminate and are maintained for long periods of time and recognize the surface antigens of recurrent tumor cells. Researchers have combined the immune response in the tumor microenvironment with pTNM staging system to construct the TNM-I (TNM-immune) staging system and for guiding postoperative treatment of colon cancer, which has proved a reliable method for clinicians [11, 12]. However, in GC with higher heterogeneity, the traditional immunohistochemical method is interfered by the complexity, heterogeneity, and inconsistency of regional tissue selection. The immune evaluation of peripheral blood may reduce the heterogeneity to provide an essential prognostic and potentially predictive tool [13]. Our previous studies have found that peripheral blood inflammatory factors are superior to traditional tumor markers in the early diagnosis of GC. Therefore, introduction of peripheral immune response as a biomarker to classify cancer will facilitate clinical decision-making [14].

This study retrospectively analyzed patients who underwent radical gastrectomy in the Harbin Medical University Cancer Hospital between 2000 and 2014. Patients with death from recurrence >5 years after radical gastrectomy and patients with early death within 2 years from stage-I and -II GC were selected. Finally, we screened the serum immune factors related to recurrence and prognosis to construct a predictive model combined with clinicopathological characteristics.

## 2. Materials and Methods

### 2.1. Patient Characteristics

Patients who underwent radical gastrectomy were selected between October 2000 and December 2014 from the Department of Gastrointestinal Surgery of Harbin Medical University Cancer Hospital. All the patients had no tumor invasion of the surrounding tissues. The diagnosis was based on paraffin sections obtained by electronic fiber gastroscopy before surgery and confirmed postoperatively by experienced pathologists. During hospitalization, all patients underwent hematological examination, abdominal ultrasonography, electrocardiography, stomach computed tomography (CT)/magnetic resonance imaging (MRI), chest radiography, and abdominal ultrasonography, and some patients underwent positron emission tomography (PET)/computed tomography (CT) when necessary.

Inclusion criteria were as follows. Group A: the survival time after radical gastrectomy was >5 years and the patients with long-term survival were followed up for at least 7 years. Group B: patients with pTNM stage-I and -II GC were followed up at least for 2 years (including loss to follow-up). This study only focused on the recurrence of patients in two years and patients in Group B will be further followed up and treated. The exclusion criteria were as follows: (1) preoperative radiotherapy and/or chemotherapy; (2) antiplatelet therapy within the previous 3 months; (3) steroid therapy upon admission; (4) recurrent GC; (5) severe heart disease; (6) hematological malignancies, including multiple myeloma; (7) complications of abdominal infection or systemic infectious disease; and (8) distant metastases. The standard postoperative chemotherapy was according to the NCCN Clinical Practice Guidelines in Oncology. The clinicopathological data were saved in the Gastric Cancer Information Management System v1.2 of Harbin Medical University Cancer Hospital (Copyright no. 2013SR087424, http:www.sgihmu.com), including sex, age, BMI, Borrmann type, tumor location, tumor diameter, pTNM stage, scope of gastrectomy, lymph node metastasis, and histological type. The pTNM stage was according to the 8th edition American Joint Committee on Cancer (AJCC). All patients were reexamined by ultrasound, CT and gastroscopy, and tumor markers at least once a year, and PET/CT was performed as needed.

### 2.2. Laboratory Examination

Complete blood count (CBC) with automated differential counts, serum fibrinogen (F), and traditional tumor markers were performed for all patients. On the day of admission or the morning of day, 2.2 ml peripheral fasting blood was collected from the cubital vein and the serum was separated for analysis. CBC was performed within 4 h. For the inflammatory index, neutrophil–lymphocyte ratio (NLR), platelet–lymphocyte ratio (PLR), and systemic immune inflammation (SII) = neutrophil count × platelet count/lymphocyte count.

### 2.3. Statistical Analysis

Overall survival (OS) was defined as the time from surgery to death from any cause due to GC. If patients were alive at last follow-up, they were censored. Log-rank test and Kaplan–Meier method were used to analyze the survival curves. The survival time was presented as median ± standard deviation. The survival analysis of Group A only included patients who survived >5 years, so the starting point was 60 months. The prognostic significance of NLR, PLR, SII, and F for patients with GC was calculated and compared according to receiver operating characteristic (ROC) curve analysis. The area under the curve (AUC) was calculated, and the optimal cut-off value was analyzed by the Youden index. The chi-square test also was used to analyze the association between blood test index and clinicopathological characteristics. *P* < 0.05 was considered statistically significant. Univariate and multivariate analyses based on cox regression were used to analyze the independent risk factors for prognosis. The variables with *P* < 0.05 in the univariate analysis were subsequently included in the multivariate analysis, and variables with *P* < 0.05 in the multivariate analysis were considered to be independent risk factors for prognosis. Odds ratios (ORs) and 95% confidence intervals (CIs) were estimated for each factor. R Studio was used to construct the nomogram model of risk assessment using the SvyNom and rms packages. The relation curve and scatter plot were drawn by GraphPad Prism 8. SPSS version 25.0 (Chicago, IL, USA) was used for analysis.

## 3. Results

### 3.1. Clinical Characteristics

**Figure 1:**
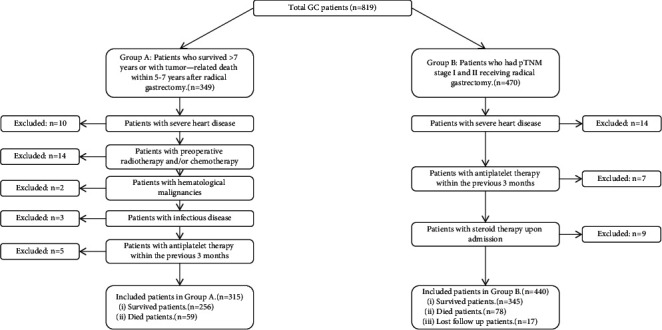
The flowchart for the process for inclusion of patients.

A total of 755 patients were selected in this retrospective research, and the process for inclusion of patients is shown schematically in Figure 1. According to the inclusion criteria, patients who survived >7 years or those with tumor-related death within 5–7 years after radical gastrectomy were designated Group A. Patients with pTNM stages I and II receiving radical gastrectomy were designated Group B. There were 315 patients (238 male, 77 female) in Group A, with a median age of 60 years, and the number with stage-I, -II, and -III GC was 91, 103, and 121, respectively. There were 440 patients (335 male, 105 female) in Group B, with a median age of 58 years, and the number with stage-I and -II GC was 160 and 280, respectively. The basic clinicopathological features of the two groups are shown in Table 1.

### 3.2. NLR, PLR, SII, F, NLR-PLR, and F-SII Score for Risk of Death

**Figure 2:**
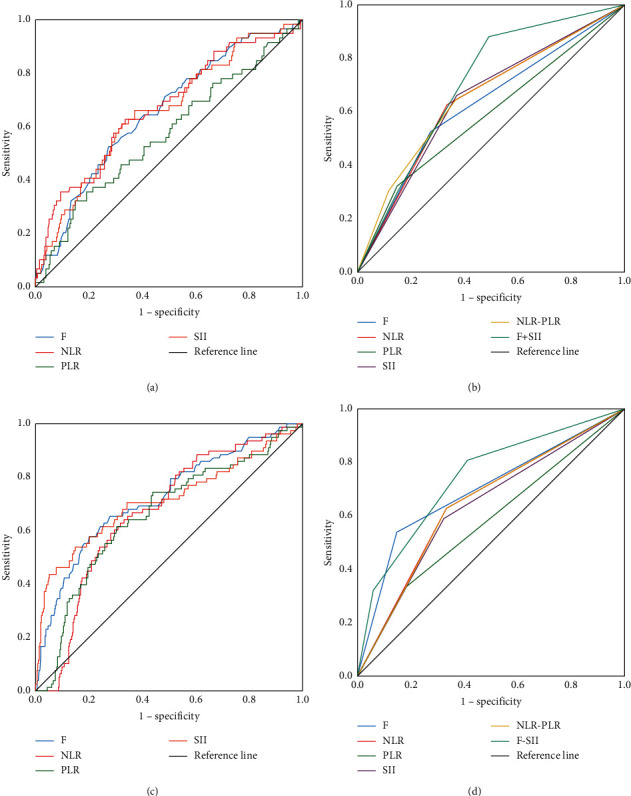
(a) ROC curve of F NLR, PLR, and SII in Group A. (b) ROC curve of F NLR, PLR, SII, NLR–PLR, and F–SII in Group A based on the cut-off threshold. (c) ROC curve of F NLR, PLR, and SII in Group B. (d) ROC curve of F NLR, PLR, SII, NLR–PLR, and F–SII in Group B based on the cut-off threshold.

According to the ROC analysis of patients in Group A, the AUC of NLR, PLR, SII, and F were 0.669 (95% CI: 0.590–0.749), 0.572 (95% CI: 0.487–0.656), 0.657 (95% CI: 0.597–0.735), and 0.654 (95% CI: 0.577–0.730), respectively (Figure 2(a)). The AUC of NLR, PLR, SII, and F in Group B were 0.670 (95% CI: 0.608–0.732), 0.658 (95% CI: 0.590–0.726), 0.712 (95% CI: 0.638–0.786), and 0.724 (95% CI: 0.656–0.787), respectively (Figure 2(c)).

We calculated the maximum Youden index of Group A as the cut-off values of these serum biomarkers. 2.13, 192.61, 495.34, and 3.36 were defined as the cut-off values of NLR, PLR, SII, and F, which were used to construct the NLR–PLR and F–SII scoring systems. In two scoring systems, patients with NLR and PLR or F and SII lower than the cut-off scored 0, patients with NLR and PLR or F and SII higher than the cut-off threshold scored 2, and other patients scored 1. The AUC of NLR, PLR, SII, F, NLR–PLR, and F–SII in Group A were 0.646 (95% CI: 0.567–0.724), 0.587 (95% CI: 0.502–0.672), 0.645 (95% CI: 0.567–0.723), 0.626 (95% CI: 0.544–0.708), 0.657 (95% CI: 0.577–0.737), and 0.703 (95% CI: 0.636–0.770), respectively (Figure 2(b)). To verify the sensitivity of this scoring system in predicting the risk of cancer mortality, we analyzed the scoring system constructed with the same cut-off value in Group B. The AUC of NLR, PLR, SII, F, NLR–PLR, and F–SII in Group B were 0.647 (95% CI: 0.575–0.720), 0.576 (95% CI: 0.499–0.653), 0.633 (95% CI: 0.560–0.707), 0.696 (95% CI: 0.622–0.769), 0.646 (95% CI: 0.574–0.718), and 0.740 (95% CI: 0.674–0.806), respectively (Figure 2(d)). This result indicated that F and SII may have wide application in predicting the risk of GC-related mortality.

### 3.3. Relationship between Serum Biomarkers and Clinicopathological Characteristics

**Figure 3:**
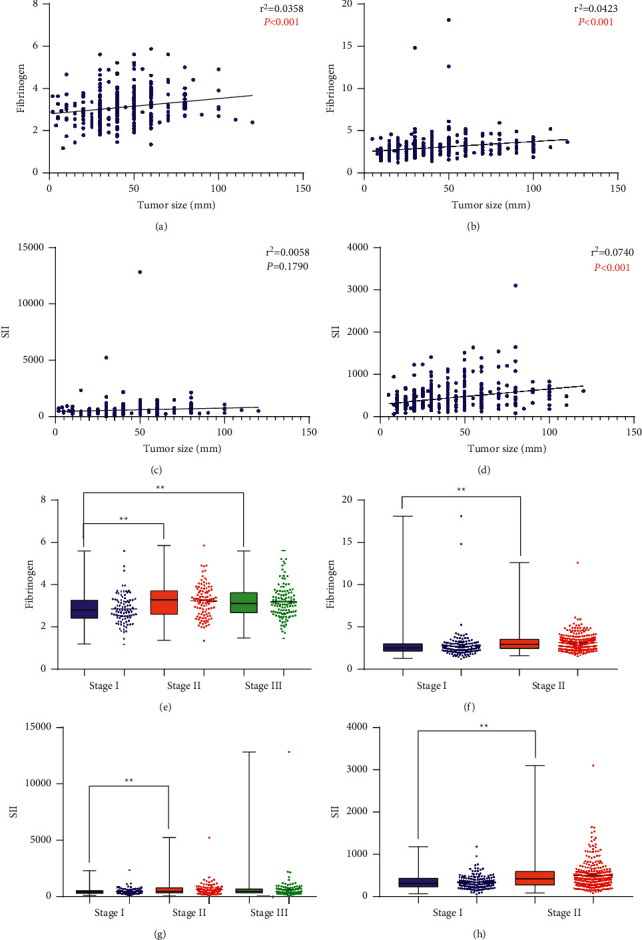
(a) Connection between F and tumor diameter in Group A. (b) Connection between F and tumor diameter in Group B. (c) Connection between SII and tumor diameter in Group A. (d) Connection between SII and tumor diameter in Group B. (e) Difference in F based on pTNM stage in Group A. (f) Difference in F based on pTNM stage in Group B. (g) Difference in SII based on pTNM stage in Group A. (h) Difference in SII based on pTNM stage in Group B. Pearson's correlation coefficient was applied to test for correlation between groups.

In Group A, F was positively correlated with tumor diameter (*r*^2^ = 0.0358, *P* < 0.001) (Figure 3(a)). F was significantly higher in stage-II GC (3.23 ± 0.77) and stage-III GC (3.20 ± 0.80) than stage-I GC (2.85 ± 0.71) (*P* < 0.05) (Figure 3(e)). SII had no correlation with tumor diameter (*r*^2^ = 0.0058, *P*=0.1790) (Figure 3(c)). Patients with stage-II GC (616.88 ± 570.10) had a significantly higher SII than patients with stage-I GC (459.96 ± 281.18) (*P* < 0.05) (Figure 3(g)). In Group B, F was also positively correlated with tumor diameter (*r*^2^ = 0.0423, *P* < 0.001) (Figure 3(b)). Patients with stage-II GC (3.14 ± 1.01) had significantly higher F than patients with stage-I GC (2.76 ± 1.68) (*P* < 0.05) (Figure 3(f)). SII was positively correlated with tumor diameter (*r*^2^ = 0.0740, *P* < 0.001) (Figure 3(d)). Patients with stage-II GC (498.25 ± 331.88) had significantly higher SII than patients with stage-I GC (342.44 ± 159.26) (*P* < 0.05) (Figure 3(h)).

### 3.4. Survival Analysis of F-SII Score System

**Figure 4:**
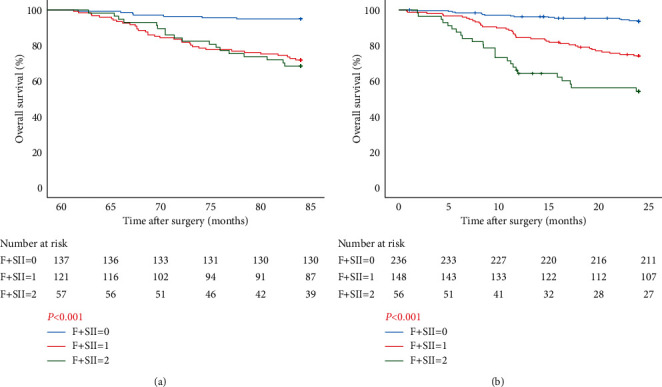
(a) Survival curves of patients in Group A based on F–SII score. (b) Survival curves of patients in Group B based on F–SII score. *P* value was calculated according to log-rank test.

According to the F–SII score system in Group A, patients with score 1 or 2 had no significant difference in OS, which was worse than that in patients with score 0. The OS of patients with scores 0, 1, and 2 was 84.0 ± 3.47, 84.0 ± 6.83, and 84.0 ± 6.18 months and the 5–7-year survival rate was 94.9%, 71.9%, and 68.4%, respectively (Figure 4(a)). F–SII score had a significant association with tumor diameter and pTNM stage (*P* < 0.001 and *P*=0.002) (Table 2). In Group B, the F–SII score was negatively correlated with OS and patients with score 0 had the best survival. The OS of patients with scores 0, 1, and 2 was 24.0 ± 4.03, 24.0 ± 6.08, and 20.52 ± 7.88 months and the 2-year survival rate was 89.4%, 72.3%, and 48.2%, respectively (Figure 4(b)). F–SII scoring system had a significant association with age, Borrmann type, tumor diameter, and pTNM stage (*P*=0.024, *P*=0.042, *P* < 0.001, and *P* < 0.001) (Table 2).

### 3.5. Univariate and Multivariate Regression Analyses

To identify the independent risk factors for prognosis in Groups A and B, univariate and multivariate analyses based on the cox regression model were performed. According to univariate analysis, age (*P*=0.027, *P* < 0.001), F (*P* < 0.001, *P*=0.001), SII (*P*=0.002, *P* < 0.001), tumor diameter (*P*=0.001, *P* < 0.001), scope of gastrectomy (*P*=0.015, *P* < 0.001), and pTNM stage (*P* < 0.001, *P* < 0.001) were significantly associated with prognosis in both groups. BMI (*P*=0.016), Borrmann type (*P*=0.014), and tumor location (*P* < 0.001) were only significant in Group B. According to multivariate analyses, F (*P*=0.031), SII (*P*=0.039), and pTNM stage (*P*=0.006) were independent risk factors for prognosis in Group A, and age (*P* < 0.001), F (*P*=0.018), SII (*P* < 0.001), Borrmann type (*P*=0.043), and scope of gastrectomy (*P*=0.035) were independent risk factors for prognosis in Group B (Table 3).

### 3.6. Construction of Nomogram for Predicting Survival

**Figure 5:**
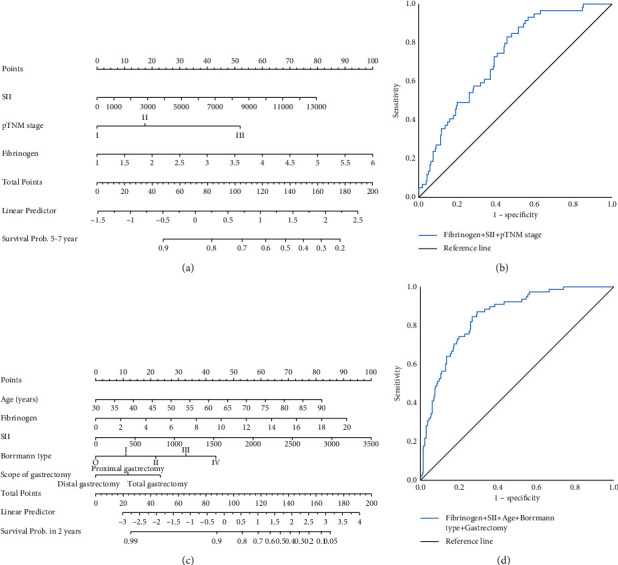
(a) Nomogram model predicting survival probability in Group A. (b) ROC curve of nomogram model in Group A. (c) Nomogram model predicting survival probability of patients in Group B. (d) ROC curve of nomogram model in Group B.

Nomogram models in predicting the prognosis of patients were constructed based on the independent risk factors for prognosis in Groups A and B (Figures 5(a)5(c)). The AUC of the model in predicting prognosis within 5–7 years after radical gastrectomy in Group A was 0.726 (95% CI: 0.662–0.790), the sensitivity was 83.1%, and the specificity was 54.3% (Figure 5(b)). The AUC of the model in predicting stage-I and -II GC patients prognosis within 2 years after radical gastrectomy in Group B was 0.848 (95% CI: 0.805–0.891), the sensitivity was 87.2%, and the specificity was 70.7% (Figure 5(d)).

## 4. Discussion

In clinical practice, patients with a survival time >5 years are often considered not to need further follow-up and medical treatment, but there are still cases of recurrence and metastasis many years after tumor resection. According to Moon et al., the recurrence rate after long-term survival is about 10% [6]. In this study, the 5–7-year mortality rate due to GC was 18.7%. The high mortality rate may be related to the lack of follow-up after 5 years in our hospital, but it is also enough to show that clinicians should pay more attention to patients with a high risk of recurrence after 5 years. As early as 1982, Koga et al. [7] reported that 5.1% of patients had recurrence after 5 years of survival despite radical gastrectomy. Lee et al. [15] proposed that pathological stage T4a was a factor for predicting late recurrence and suggested that patients in T4a stage should be followed up and reexamined for longer after surgery.

This study proposes another viewpoint that may solve this problem. We found that F, SII, and pTNM stages were independent risk factors for death of GC patients 5–7 years after radical gastrectomy and could be used to construct a prognostic prediction model. The AUC was 0.726, the sensitivity was 83.1%, and the specificity was 54.3%. For similar studies, Katai et al. [8] pointed out that patients with a longer time between recurrence and surgery often had deeper infiltration of the primary tumor, and the main recurrence was always peritoneal metastasis. In our study, 59 patients died >5 years after surgery, and pT1 was seen in two cases (3.4%), pT2 in seven (11.9%), pT3 in seven (11.9%), and pT4 in 43 (72.9%). Huang et al. [16] reported that F could combine with carbohydrate antigen (CA)125 and CA19-9 to predict the risk of peritoneal metastasis in patients with GC after surgery. Recurrence after long-term survival may be related to the mechanism of tumor dormancy [17, 18]. Early postoperative recurrence and metastatic dormancy are a particular mode of recurrence of malignant tumors. When the tumor cells break through the mechanical pressure and immune defense, they can enter the peripheral blood through the vascular endothelium and transform into circulating tumor cells (CTCs). However, some CTCs cannot adapt to the new microenvironment and the lack of adhesive factors and key signaling molecules can lead to tumor dormancy. With a change in the immune microenvironment of the tumor dormancy site, or another unknown stimulation, activated tumor cells can convert to epithelial mesenchymal transition (EMT) through Smad2- and *β*-catenin-related signaling pathways and develop powerful invasiveness and rapid proliferation, penetrate vascular tissue, and even progress to distant metastasis. In the present study, patients with high F–SII score had a significantly increased risk of distant dormant metastatic lesions, which should be paid more attention during postoperative follow-up and receive additional treatment [19, 20].

With increased awareness of cancer prevention and improvement of detection rate of early GC through gastroscopy in recent years, the proportion of GC patients with early GC in developed countries such as South Korea has increased to 57.7%, and patients with stage-I and -II GC in Japan account for 72.3% [21–23]. According to epidemiological statistics, the recurrence rates of patients with stage-I and -II GC are about 10% and 30%, respectively [24]. At present, radical surgery, endoscopic mucosal resection (EMR), and endoscopic submucosal dissection (ESD) are alternative treatments for patients with GC invading the mucosa and submucosa [25–27]. However, developed countries such as South Korea and Japan are more inclined to ESD and EMR, while for developing countries in Southeast Asia and China, radical surgery is a more common choice. This phenomenon may be due to the inevitable bleeding complications and residual tumor in gastrointestinal endoscopy in developing countries [28]. At the same time, these surgeons always choose radical resection of GC to avoid early occurrence of metastasis because it is difficult to find micrometastases by general imaging examination. With the increase in the proportion of patients with stage-I and -II GC, more attention should be paid to improving evaluation of these patients, along with appropriate treatment methods and prognosis of recurrence.

In GC, an early stage is associated with long disease-free survival and OS, in addition to low risk of relapse and metastasis, which leads to neglect of their follow-up and treatment in clinical practice. Therefore, there is an urgent requirement to find more sensitive markers for GC at an early clinical stage. To supplement the current staging system, we constructed a predictive model by screening for serum immune factors and combining with clinicopathological features. We found that F, SII, age, Borrmann type, and scope of gastrectomy were independent risk factors associated with the 2-year survival probability of stage-I and -II GC. The AUC of the constructed nomogram was 0.848, the sensitivity was 87.2%, and the specificity was 70.7%. Previous studies have found that infiltration of lymphocytes and macrophages into tumor tissues increases significantly when gastric intraepithelial neoplasia develops into early GC [29]. This particular tumor environment also indirectly affects the immune status of the peripheral blood. For patients receiving proximal gastrectomy, the probability of postoperative acid reflux is significantly increased [30]. Patients receiving total gastrectomy also show poor prognosis due to 5%–19% weight loss and malnutrition [31, 32]. Therefore, the selection of surgical approach for GC will significantly affect the quality of life and survival rate, which needs to be carefully considered. According to SEER Medicare database, only 72.4% of patients with stage-IB GC and 50.6% of patients with stage-II GC received surgical treatment [33]. Therefore, we suggest that nutritional support, follow-up observation, and adjuvant chemoradiotherapy should be emphasized in patients with high nomogram model score after surgery. Similar conclusions have been confirmed by Datta et al. [34], who found significantly improved survival probability of patients with stage-IB and -II GC. In our study, high F–SII score indicated a high risk of early invisible metastasis, and more attention should be paid to improving evaluation of these patients.

Another major concern is the impact of *Helicobacter pylori* on the occurrence and development of GC. Since this study is a retrospective analysis, most patients admitted to the hospital for surgical treatment were not routinely tested for *H. pylori*, which led to the missing data. However, it is undeniable that *H. pylori* plays an important role in peripheral blood immunity, tumor microenvironmental immune infiltration, and tumor progression [35]. According to data in the literature, approximately 1% to 3% of infected patients in the world will develop GC, while the proportion of noninfected patients is 0.13% [36]. For GC patients combined with *H. pylori* infection, anti-*H. pylori* treatment should be performed after surgery. A recent study pointed out that clarithromycin and levofloxacin have better anti-*H. pylori* efficacy. However, high BMI has been shown to be associated with high eradication failure rates [37]. Therefore, the weight management should start after surgery, based on guidelines for lipid screening and blood pressure determination. Moreover, allium vegetables and their components have recently been intensively studied in digestive system tumors. Allium and its constituents have anti-inflammatory, immunomodulating, and anticancer effects, which have been observed in many in vivo and in vitro experiments [38]. These substances may be used as an early prevention of the recurrence of GC, which may reduce the morbidity and mortality risks to a certain extent, while at the same time being more cost-effective. Therefore, in addition to traditional drug treatment, postoperative patients should also be given dietary guidance.

In this study, we found that F and SII were independent risk factors for predicting prognosis and mortality whether in the early or subsiding stage of GC, which shared some similarities with our previous study [39]. The similarities between the two conclusions should be verified. Neutrophils, lymphocytes, and platelets are likely to be important for tumor recurrence and tumor dormancy [40, 41]. F, which plays a role in coagulation, cell adhesion, and inflammation, also promotes tumor progression and distant metastasis and is considered to be a potential molecular marker of various malignant tumors [42–45]. Mucosal epithelial cells can produce F by inflammatory stimuli. F may convert to insoluble fibrin and exert a considerable influence on cancer cell progression. Similar studies have demonstrated that serum platelets and F enhance metastasis of tumor cells by impeding intravascular tumor cell clearance by natural killer (NK) cells. At the same time, platelet expression of aIIb*β*3 may interact with tumor cells through an F bridge and contribute to metastasis. In the immune response of peripheral blood, lymphocytes kill CTCs and inhibit distant metastasis. Proliferating neutrophils can secrete cytokines such as interleukin-10 and tumor necrosis factor-*α*, inhibit activity of lymphocytes (CD4^+^ and CD8^+^ T cells) and NK cells, and further promote the immune escape of CTCs. At the same time, tissue destruction and nonspecific inflammatory reaction were shown to be dependent on neutrophil activity around cancer cells. The cascade effect caused by neutrophils is considered to promote the distant implantation and metastasis of CTC.

The predictive accuracy of this traditional AJCC staging system relies on the assumption that the risk of tumor recurrence decreases with survival time, and tumor progression depends only on the characteristics of the tumor cells. Therefore, it is inevitable that this staging system pays less attention to cancer in early stage or subsiding stage. Thanks to the promotion of Real-World Research and Big Data for Cancer Research [46], we can integrate and comprehensively analyze the clinicopathological characteristics and immune status of these two groups of patients. The predictive model based on immune response, pathological stage, and clinical characteristics can predict the risk of postoperative recurrence in patients who are difficult to assess [47]. Our study was based on the clinical limitations of the TNM staging system and proposed a more accurate individualized predictive model, which is worthy of further promotion and validation in clinical practice.

This was a retrospective study that had some limitations. Firstly, this study focused on Asian patients in a single center, and whether the results are widely applicable to white or black populations needs further study. Secondly, the recurrence patterns of the two groups, such as primary recurrence, peritoneal recurrence, or distant metastasis and *H. pylori* infection, could not be completely recorded, which also needs to be explored in future research.

## 5. Conclusion

In summary, we developed predictive models based on F and SII combined with clinicopathological features to predict early mortality of stage-I and -II GC, as well as patients with cancer regression. This study suggests that radical resection and appropriate postoperative treatment can be performed for patients with high probability of recurrence of stage-I and -II GC. Further follow-up and imaging examination should be carried out for patients with high probability of recurrence whose survival time is > 5 years. At the same time, proper weight management and dietary guidance are also essential for patients.

## Figures and Tables

**Table 1:** Baseline characteristics of the patient in Groups A and B.

Characteristics	Group A (*n* = 315)	Group B (*n* = 440)
Sex		
Male	238 (75.6)	335 (76.1)
Female	77 (24.4)	105 (23.9)

Age (years)		
≤60	160 (50.8)	257 (58.4)
>60	155 (49.2)	183 (41.6)

BMI		
≤21.92	145 (46.0)	168 (38.2)
>21.92	119 (37.8)	272 (61.8)
Unknown	51 (16.2)	0

Borrmann type		
0	20 (6.3)	60 (13.6)
1	14 (4.4)	23 (5.2)
2	75 (23.8)	128 (29.1)
3	194 (61.6)	210 (47.7)
4	12 (3.8)	19 (4.3)

Tumor location		
Upper third	31 (9.8)	46 (10.5)
Middle third	57 (18.1)	53 (12.0)
Lower and entire third	227 (72.1)	341 (77.5)

Tumor diameter (mm)		
≤50	232 (73.7)	337 (76.6)
>50	83 (26.3)	103 (23.4)

Scope of gastrectomy		
Proximal gastrectomy	27 (8.6)	39 (8.9)
Distal gastrectomy	232 (73.7)	342 (77.7)
Total gastrectomy	56 (17.8)	59 (13.4)

Lymph node metastasis rate (%)		
0	153 (48.6)	326 (74.1)
>0 to ≤0.3	114 (36.2)	108 (24.5)
>0.3 to ≤1.0	48 (15.2)	6 (1.4)

pTNM stage^a^		
I	91 (28.9)	160 (36.4)
II	103 (32.7)	280 (63.6)
III	121 (38.4)	—

Histological type		
Well and moderately differentiated	121 (38.4)	208 (47.3)
Poorly differentiated	168 (53.3)	180 (40.9)
Others	26 (8.3)	52 (11.8)

*H. pylori* infection		
Negative	43 (13.7)	46 (10.5)
Positive	60 (19.0)	72 (16.4)
Unknown	212 (67.3)	322 (73.2)

^a^Based on the eighth edition of the AJCC Cancer Staging Manual of the American Joint Committee on Cancer.

**Table 2:** The chi-square analysis of the connection between F–SII and clinicopathological features in Groups A and B.

Characteristics	Group A				Group B			
	F–SII = 0 (*n* = 137)	F–SII = 1 (*n* = 121)	F–SII = 2 (*n* = 57)	*P* value	F–SII = 0 (*n* = 236)	F–SII = 1 (*n* = 148)	F–SII = 2 (*n* = 56)	*P* value
Sex				0.420				0.120
Male	99 (41.6)	93 (39.1)	46 (19.3)		187 (55.8)	104 (31.0)	44 (13.1)	
Female	38 (49.4)	28 (36.4)	11 (14.3)		49 (46.7)	44 (41.9)	12 (11.4)	
Age (years)				0.224				**0.024**
≤60	76 (47.5)	60 (37.5)	24 (15.0)		150 (58.4)	82 (31.9)	25 (9.7)	
>60	61 (39.4)	61 (39.4)	33 (21.3)		86 (47.0)	66 (36.1)	31 (16.9)	
BMI				0.133				0.083
≤21.92	72 (49.7)	46 (31.7)	27 (18.6)		81 (48.2)	59 (35.1)	28 (16.7)	
>21.92	48 (40.3)	52 (43.7)	19 (16.0)		155 (57.0)	89 (32.7)	28 (10.3)	
Borrmann type				0.145				**0.042**
0	14 (70.0)	5 (25.0)	1 (5.0)		43 (71.7)	16 (26.7)	1 (1.7)	
1	6 (42.9)	5 (35.7)	3 (21.4)		15 (65.2)	7 (30.4)	1 (4.3)	
2	36 (48.0)	25 (33.3)	14 (18.7)		62 (48.4)	46 (35.9)	20 (15.6)	
3	79 (40.7)	78 (40.2)	37 (19.1)		108 (51.4)	72 (34.3)	30 (14.3)	
4	2 (16.7)	8 (66.7)	2 (16.7)		8 (42.1)	7 (36.8)	4 (21.1)	
Tumor location				0.639				0.189
Upper third	13 (41.9)	14 (45.2)	4 (12.9)		20 (43.5)	20 (43.5)	6 (13.0)	
Middle third	24 (42.1)	25 (43.9)	8 (14.0)		23 (43.4)	23 (43.4)	7 (13.2)	
Lower and entire third	100 (44.1)	82 (36.1)	45 (19.8)		193 (56.6)	105 (30.8)	43 (12.6)	
Tumor diameter (mm)				**＜0.001**				**＜0.001**
≤50	119 (51.3)	73 (31.5)	40 (17.2)		205 (60.8)	101 (30.0)	31 (9.2)	
>50	18 (21.7)	48 (57.8)	17 (20.5)		31 (30.1)	47 (45.6)	25 (24.3)	
Scope of gastrectomy				0.739				0.151
Proximal gastrectomy	12 (44.4)	11 (40.7)	4 (14.8)		18 (46.2)	15 (38.5)	6 (15.4)	
Distal gastrectomy	105 (45.3)	85 (36.6)	42 (18.1)		194 (56.7)	106 (31.0)	42 (12.3)	
Total gastrectomy	20 (35.7)	25 (44.6)	11 (19.6)		24 (40.7)	27 (45.8)	8 (13.6)	
Lymph node metastasis rate (%)				0.072				0.790
0	69 (45.1)	56 (36.6)	28 (18.3)		176 (54.0)	111 (34.0)	39 (12.0)	
>0 to ≤0.3	55 (48.2)	38 (33.3)	21 (18.4)		58 (53.7)	34 (31.5)	16 (14.8)	
>0.3 to ≤1	13 (27.1)	27 (56.3)	8 (16.7)		2 (33.3)	3 (50.0)	1 (16.7)	
pTNM stage^a^				**0.002**				**＜0.001**
I	53 (58.2)	29 (31.9)	9 (9.9)		117 (73.1)	38 (23.8)	5 (3.1)	
II	40 (38.8)	36 (35.0)	27 (26.2)		119 (42.5)	110 (39.3)	51 (18.2)	
III	44 (36.4)	56 (46.3)	21 (17.4)		-	-	-	
Histological type				0.320				0.966
Well and moderately differentiated	56 (46.3)	43 (35.5)	22 (18.2)		110 (52.9)	70 (33.7)	28 (13.5)	
Poorly differentiated	73 (43.5)	63 (37.5)	32 (19.0)		97 (53.9)	60 (33.3)	23 (12.8)	
Others	8 (30.8)	15 (57.7)	3 (11.5)		29 (55.8)	18 (34.6)	5 (9.6)	
*H. pylori* infection				0.983				0.781
Negative	17 (39.5)	15 (34.9)	11 (25.6)		20 (43.5)	17 (37.0)	9 (19.6)	
Positive	23 (38.3)	22 (36.7)	15 (25.0)		36 (50.0)	23 (31.9)	13 (18.1)	

SII: systemic immune inflammation index, *F*: fibrinogen. ^a^Based on the eighth edition of the AJCC Cancer Staging Manual of the American Joint Committee on Cancer. Statistically significant *P* values are in bold (*P* < 0.05).

**Table 3:** Prognosis factors of patients in Groups A and B by univariate and multivariate cox regression analysis.

Characteristics	Group A				Group B			
	Univariate analysis		Multivariate analysis		Univariate analysis		Multivariate analysis	
	Or (95% CI)	*P* value	Or (95% CI)	*P* value	Or (95% CI)	*P* value	Or (95% CI)	*P* value
Sex		0.073	—	—		0.445	—	—
Male	1				1			
Female	2.009 (0.938–4.305)				1.263 (0.694–2.300)			
Age (years)	1.034 (1.004–1.066)	**0.027**	1.022 (0.989–1.056)	0.196	1.069 (1.040–1.098)	＜**0.001**	1.070 (1.037–1.105)	＜**0.001**
Fibrinogen	1.909 (1.336–2.728)	＜**0.001**	1.547 (1.041–2.300)	**0.031**	1.556 (1.201–2.017)	**0.001**	1.268 (1.042–1.544)	**0.018**
SII	1.001 (1.000–1.002)	**0.002**	1.001 (1.000–1.002)	**0.039**	1.003 (1.002–1.004)	＜**0.001**	1.003 (1.002–1.004)	＜**0.001**
BMI	0.958 (0.866–1.061)	0.412	—	—	0.897 (0.822–0.980)	**0.016**	0.911 (0.821–1.009)	0.075
Borrmann type		0.897	—	—		**0.014**		0.165
0	1				1		1	
1	0.667 (0.104–4.261)	0.668			5.619 (0.484–65.218)	0.168	2.806 (0.188–41.846)	0.454
2	1.000 (0.291–3.432)	1.000			11.579 (1.519–88.265)	**0.018**	6.647 (0.742–59.542)	0.090
3	0.943 (0.298–2.985)	0.920			17.481 (2.360–129.505)	**0.005**	9.346 (1.074–81.364)	**0.043**
4	0.364 (0.036–3.707)	0.393			27.231 (3.016–245.887)	**0.003**	12.220 (1.014–147.266)	**0.049**
Tumor location		0.342	—	—		＜**0.001**		0.844
Lower and entire third	1				1		1	
Middle third	1.619 (0.807–3.250)	0.175			2.705 (1.395–5.246)	**0.003**	1.148 (0.447–2.952)	0.774
Upper third	1.451 (0.583–3.608)	0.424			3.027 (1.520–6.029)	**0.002**	0.817 (0.227–2.949)	0.758
Tumor diameter (mm)	1.025 (1.011–1.039)	**0.001**	1.009 (0.993–1.026)	0.281	1.023 (1.012–1034)	＜**0.001**	0.996 (0.981–1.012)	0.641
Scope of gastrectomy		0.051		0.547		＜**0.001**		**0.035**
Distal gastrectomy	1		1		1		1	
Proximal gastrectomy	0.435 (0.223–0.850)	**0.015**	1.524 (0.503–4.618)	0.457	4.711 (2.311–9.605)	＜**0.001**	4.846 (1.277–18.394)	**0.020**
Total gastrectomy	0.521 (0.169–1.607)	0.257	1.435 (0.658–3.130)	0.363	2.973 (1.571–5.629)	**0.001**	2.868 (1.114–7.383)	**0.029**
Lymph node metastasis rate (%)	3.871 (0.872–17.182)	0.075	—	—	15.462 (0.938–254.878)	0.055	—	—
pTNM stage		＜**0.001**		**0.019**		＜**0.001**		0.169
I	1		1		1		1	
II	5.570 (1.834–16.918)	**0.002**	3.530 (1.117–11.156)	**0.032**	5.487 (2.656–11.332)		1.833 (0.774–4.344)	
III	8.500 (2.893–24.975)	＜**0.001**	5.161 (1.642–16.225)	**0.005**	—	—	—	—
Histological type		0.764	—	—		0.221	—	—
Well and moderately differentiated	1				1			
Poorly differentiated	1.094 (0.375–3.189)	0.870			1.588 (0.937–2.690)	0.086		
Others	0.876 (0.305–2.515)	0.806			1.413 (0.641–3.115)	0.392		
*H. pylori* infection	1.058 (0.433–2.583)	0.902	—	—	0.672 (0.301–1.499)	0.331	—	—

^a^Based on the eighth edition of the AJCC Cancer Staging Manual of the American Joint Committee on Cancer. Statistically significant *P* values are in bold (*P* < 0.05).

## Data Availability

The datasets used in this study are available from the corresponding author on reasonable request. More information can also be obtained from the Gastric Cancer Information Management System v1.2 of Harbin Medical University Cancer Hospital (copyright no. 2013SR087424, http://www.sgihmu.com/).

## References

[1] Global Burden of Disease Cancer C. (2019). Global, regional, and national cancer incidence, mortality, years of life lost, years lived with disability, and disability-adjusted life-years for 29 cancer groups, 1990 to 2017: a systematic analysis for the global burden of disease study. *JAMA Oncology*.

[2] Oh S. E., An J. Y., Choi M.-G., Lee J. H., Sohn T. S., Bae J. M. (2020). Comparisons of remnant primary, residual, and recurrent gastric cancer and applicability of the 8th AJCC TNM classification for remnant gastric cancer staging. *European Journal of Surgical Oncology*.

[3] Japanese Gastric Cancer A. (2021). Japanese gastric cancer treatment guidelines 2018. *Gastric Cancer*.

[4] Siegel R. L., Miller K. D., Jemal A. (2020). Cancer statistics, 2020. *CA: A Cancer Journal for Clinicians*.

[5] Chen W. (2013). Cancer incidence and mortality in China. *Cancer Letters*.

[6] Moon Y. W., Jeung H.-C., Rha S. Y. (2007). Changing patterns of prognosticators during 15-year follow-up of advanced gastric cancer after radical gastrectomy and adjuvant chemotherapy: a 15-year follow-up study at a single Korean institute. *Annals of Surgical Oncology*.

[7] Koga S., Kaibara N., Kishimoto H. (1982). Comparison of 5- and 10-year survival rates in operated gastric cancer patients. *Langenbecks Archiv for Chirurgie*.

[8] Katai H., au fnm, Ishikawa T. (2018). Five-year survival analysis of surgically resected gastric cancer cases in Japan: a retrospective analysis of more than 100,000 patients from the nationwide registry of the Japanese Gastric Cancer Association (2001–2007). *Gastric Cancer*.

[9] Mu G.-C., Huang Y., Liu Z.-M., Wu X.-H., Qin X.-G., Chen Z.-B. (2019). Application value of nomogram and prognostic factors of gastric cancer patients who underwent D2 radical lymphadenectomy. *BMC Gastroenterology*.

[10] Ott P. A., Hu Z., Keskin D. B. (2017). An immunogenic personal neoantigen vaccine for patients with melanoma. *Nature*.

[11] Pagès F., Mlecnik B., Marliot F. (2018). International validation of the consensus Immunoscore for the classification of colon cancer: a prognostic and accuracy study. *Lancet*.

[12] Galon J., Costes A., Sanchez-Cabo F. (2006). Type, density, and location of immune cells within human colorectal tumors predict clinical outcome. *Science*.

[13] Gemenetzis G., Bagante F., Griffin J. F. (2017). Neutrophil-to-lymphocyte ratio is a predictive marker for invasive malignancy in intraductal papillary mucinous neoplasms of the pancreas. *Annals of Surgery*.

[14] Fang T., Wang Y, Yin X (2020). Diagnostic sensitivity of NLR and PLR in early diagnosis of gastric cancer. *Journal of Immunology Research*.

[15] Lee J.-H., Kim H.-I., Kim M. G., Ha T. K., Jung M.-S., Kwon S. J. (2016). Recurrence of gastric cancer in patients who are disease-free for more than 5 years after primary resection. *Surgery*.

[16] Huang C., Liu Z., Xiao L. (2019). Clinical significance of serum CA125, CA19-9, CA72-4, and fibrinogen-to-lymphocyte ratio in gastric cancer with peritoneal dissemination. *Frontiers in Oncology*.

[17] Giancotti F. G. (2013). Mechanisms governing metastatic dormancy and reactivation. *Cell*.

[18] Aguirre-Ghiso J. A. (2007). Models, mechanisms and clinical evidence for cancer dormancy. *Nature Reviews Cancer*.

[19] Mohme M., Riethdorf S., Pantel K. (2017). Circulating and disseminated tumour cells - mechanisms of immune surveillance and escape. *Nature Reviews Clinical Oncology*.

[20] Sun Y.-F., Guo W., Xu Y. (2018). Circulating tumor cells from different vascular sites exhibit spatial heterogeneity in epithelial and mesenchymal composition and distinct clinical significance in hepatocellular carcinoma. *Clinical Cancer Research*.

[21] Kim J. W., Hwang I., Kim M.-J., Jang S. J. (2009). Clinicopathological characteristics and predictive markers of early gastric cancer with recurrence. *Journal of Korean Medical Science*.

[22] Jeong O., Park Y.-K. (2011). Clinicopathological features and surgical treatment of gastric cancer in South Korea: the results of 2009 nationwide survey on surgically treated gastric cancer patients. *Journal of Gastric Cancer*.

[23] Park J. H., Ryu M.-H., Kim H. J. (2016). Risk factors for selection of patients at high risk of recurrence or death after complete surgical resection in stage I gastric cancer. *Gastric Cancer*.

[24] Kao Y.-C., Fang W.-L., Wang R.-F. (2019). Clinicopathological differences in signet ring cell adenocarcinoma between early and advanced gastric cancer. *Gastric Cancer*.

[25] Lee D. S., Park J. K., Lee S. J., Cheon G. J. (2020). Clinical significance of regional lymph node enlargement in patients with EGC within the expanded criteria for ESD. *BMC Gastroenterology*.

[26] Chung I.-K., Lee J. H., Lee S.-H. (2009). Therapeutic outcomes in 1000 cases of endoscopic submucosal dissection for early gastric neoplasms: Korean ESD Study Group multicenter study. *Gastrointestinal Endoscopy*.

[27] Zhao Y., Wang C. (2018). Long-term clinical efficacy and perioperative safety of endoscopic submucosal dissection versus endoscopic mucosal resection for early gastric cancer: an updated meta-analysis. *BioMed Research International*.

[28] Zhou Y., Zhou S., Shi Y., Zheng S., Liu B. (2019). Endoscopic submucosal dissection for gastric ectopic pancreas: a single-center experience. *World Journal of Surgical Oncology*.

[29] Zhang Y., Wu X., Zhang C. (2020). Dissecting expression profiles of gastric precancerous lesions and early gastric cancer to explore crucial molecules in intestinal‐type gastric cancer tumorigenesis. *The Journal of Pathology*.

[30] Golematis B., Misitzis J., Yiannitsiotis A., Papachristou D. N., Delicaris P. (1982). Total colectomy with resection of rectal mucosa and ileo-anal implantation for ulcerative colitis. *South African Journal of Surgery*.

[31] Tokunaga M., Hiki N., Fukunaga T., Ohyama S., Yamaguchi T., Nakajima T. (2009). Better 5-year survival rate following curative gastrectomy in overweight patients. *Annals of Surgical Oncology*.

[32] Feng F., Zheng G., Guo X. (2018). Impact of body mass index on surgical outcomes of gastric cancer. *BMC Cancer*.

[33] Snyder R. A., Penson D. F., Ni S., Koyama T., Merchant N. B. (2014). Trends in the use of evidence-based therapy for resectable gastric cancer. *Journal of Surgical Oncology*.

[34] Datta J., McMillan M. T., Ruffolo L. (2016). Multimodality therapy improves survival in resected early stage gastric cancer in the United States. *Annals of Surgical Oncology*.

[35] Charitos I. A., D’Agostino D., Topi S., Bottalico L. (2021). 40 Years of *Helicobacter pylori*: a revolution in biomedical thought. *Gastroenterology Insights*.

[36] WHO (2020). *WHO Report on Cancer: Setting Priorities, Investing Wisely and Providing Care for All*.

[37] Mohammed S. A. (2019). Clarithromycin versus levofloxacin-based regimens for Helicobacter pylori eradication in the kurdistan region of Iraq: a randomized clinical trial. *Gastroenterology Insights*.

[38] Forma A., Chilimoniuk Z., Januszewski J., Sitarz R. (2021). The potential application of allium extracts in the treatment of gastrointestinal cancers. *Gastroenterology Insights*.

[39] Xin Y. (2021). Prognostic significance of serum inflammation indexes in different Lauren classification of gastric cancer. *Cancer Medicine*.

[40] Nagtegaal I. D., Marijnen C. A., Kranenbarg E. K. (2001). Local and distant recurrences in rectal cancer patients are predicted by the nonspecific immune response; specific immune response has only a systemic effect - a histopathological and immunohistochemical study. *BMC Cancer*.

[41] Zhang D., Zhou J., Tang D. (2017). Neutrophil infiltration mediated by CXCL5 accumulation in the laryngeal squamous cell carcinoma microenvironment: a mechanism by which tumour cells escape immune surveillance. *Clinical Immunology*.

[42] Desgrosellier J. S., Cheresh D. A. (2010). Integrins in cancer: biological implications and therapeutic opportunities. *Nature Reviews Cancer*.

[43] Lee S. E., Lee J. H., Ryu K. W. (2012). Preoperative plasma fibrinogen level is a useful predictor of adjacent organ involvement in patients with advanced gastric cancer. *Journal of Gastric Cancer*.

[44] Sheng L., Luo M., Sun X., Lin N., Mao W., Su D. (2013). Serum fibrinogen is an independent prognostic factor in operable nonsmall cell lung cancer. *International Journal of Cancer*.

[45] Bloomston M., Zhou J. X., Rosemurgy A. S., Frankel W., Muro-Cacho C. A., Yeatman T. J. (2006). Fibrinogen *γ* overexpression in pancreatic cancer identified by large-scale proteomic analysis of serum samples. *Cancer Research*.

[46] Peng W., Zhang F., Wang Z. (2020). Large scale, multicenter, prospective study of apatinib in advanced gastric cancer: a real-world study from China. *Cancer Management and Research*.

[47] Liu X., Wu Z., Lin E. (2019). Systemic prognostic score and nomogram based on inflammatory, nutritional and tumor markers predict cancer-specific survival in stage II-III gastric cancer patients with adjuvant chemotherapy. *Clinical Nutrition*.

